# ETS-1 in tumor immunology: implications for novel anti-cancer strategies

**DOI:** 10.3389/fimmu.2025.1526368

**Published:** 2025-03-20

**Authors:** SiYu Wang, Lei Wan, XiaoJun Zhang, HaoXiang Fang, MengYu Zhang, Feng Li, DaWei Yan

**Affiliations:** ^1^ Department of Rheumatology and Immunology, Anhui University of Chinese Medicine First Clinical Medical College, Hefei, Anhui, China; ^2^ Department of Rheumatology and Immunology, The First Affiliated Hospital of Anhui University of Chinese Medicine, Hefei, Anhui, China; ^3^ Academic Affairs Office, Anhui University of Chinese Medicine, Hefei, Anhui, China

**Keywords:** ETS family, tumor immunology, ETS-1, biological functions, immune microenvironment, immunotherapy targets

## Abstract

ETS-1, a key member of the Erythroblast Transformation-Specific (ETS) transcription factor family, plays an important role in cell biology and medical research due to its wide expression profile and strong transcriptional regulation ability. It regulates fundamental biological processes, including cell proliferation, differentiation, and apoptosis, and is involved in tumorigenesis and metastasis, promoting malignant behaviors such as angiogenesis, matrix degradation, and cell migration. Given the association between ETS-1 overexpression and the aggressive characteristics of multiple malignancies, it represents a promising therapeutic target in cancer treatment. This study aims to systematically analyze the role of ETS-1 within the tumor immune microenvironment, elucidating its mechanisms in cancer initiation, progression, and metastasis. It also investigates the differential expression of ETS-1 across tumor tissues and adjacent normal tissues, exploring its potential as a molecular marker for tumor diagnosis and prognosis.

## 1 Introduction

The Erythroblast Transformation-Specific (ETS) transcription factor family consists of proteins characterized by a highly conserved ETS domain, which is involved in key biological processes, including cell proliferation, differentiation, apoptosis, and organogenesis (1, 2). This family has numerous members with coding genes distributed across various genomic loci, regulating downstream gene expression through specific DNA sequence recognition and binding to gene promoters or enhancers (3–5). ETS transcription factors are important in embryonic development, tissue homeostasis, and disease progression, making them one of the hot spots in biological and medical research (6).

Among the ETS family, ETS-1 is characterized by its broad expression and potent transcriptional regulation, making it a key research subject (7). ETS-1 participates in lymphocyte development and differentiation and significantly influences the proliferation, migration, and invasion of various cell types (8, 9). Notably, aberrant ETS-1 expression in various malignancies is strongly associated with tumor progression, including angiogenesis, extracellular matrix degradation, and metastasis—crucial steps in cancer invasion (10–13). Therefore, ETS-1 is considered a promising therapeutic target in cancer treatment, and its comprehensive study holds significant scientific and clinical importance.

Research in tumor immunity is a prominent focus in biomedicine, aiming to elucidate the mechanisms of interaction between tumors and the immune system and to identify effective immunotherapy strategies. Advances in understanding the tumor immune microenvironment have highlighted the immune system’s vital role in tumorigenesis, progression, and metastasis. Immune cells not only inhibit tumor growth through direct cytotoxicity but also modulate the tumor microenvironment *via* cytokine secretion (14). Therefore, a detailed investigation into the roles of key molecules such as ETS-1 in tumor immunity is of great significance for developing novel immunotherapeutic approaches.

This review systematically examines the expression and function of ETS-1 within the tumor immune microenvironment, aiming to elucidate its mechanisms in tumor initiation, progression, and metastasis. To thoroughly understand ETS-1’s role, we compare its expression in various tumor types and adjacent normal tissues. Evaluating the differential expression of ETS-1 across cancer subtypes may facilitate the identification of novel molecular markers for tumor diagnosis and prognosis. Moreover, this study explores the relationship between the molecular structure of ETS-1 and its functional roles. As a transcription factor, ETS-1’s activity is determined by key domains, including the ETS domain, transcriptional activation domain, and protein interaction domain. This review analyzes the binding mechanisms of ETS-1 to target gene promoter DNA using molecular biology and biochemical methods, thereby revealing the molecular basis of its transcriptional regulation. It also explores the interactions between ETS-1 and other transcription factors or signaling molecules to clarify its involvement in complex networks of tumor immune regulation.

Overall, this review aims to provide a comprehensive analysis of ETS-1’s mechanisms in tumor immunity, offering novel theoretical and experimental insights for tumor diagnosis, treatment, and prognosis assessment.

## 2 Overview of ETS transcription factor family

### 2.1 ETS family members and classification

The ETS transcription factor family is a large and diverse gene family that is present in humans and various other species (1). The family members are further subdivided into multiple subfamilies based on sequence similarity and functional differences of the ETS domain (15–17). As genomics research advances, more ETS family members continue to be identified. The classification criteria include the conserved sequences within the ETS domain, structural protein features, combinations of functional domains, and the types of target genes they regulate.

Each ETS subfamily has unique structural and functional characteristics. For instance, the Polymoma Virus Enhancer-3 (PEA3) subfamily (e.g., PEA3, ETS Variant Transcription Factor 4 (E1AF), and Erythromycin Resistance Methylases (ERM)) is involved in processes such as cell migration, invasion, and tumor progression (18–20). The ETS Related Gene (ERG) subfamily is closely associated with heart and vascular development (21), while the ETV6 (or TEL) subfamily (e.g., TEL and ETS variant 6 (ETV6)) plays a role in hematopoietic system development and tumorigenesis (22). Furthermore, other subfamilies, such as ETS variant (ETV), friend leukemia virus integration (FLI), and GA binding protein transcription factor subunit alpha (GABPA), are implicated in a wide range of biological functions, including cell proliferation, differentiation, metabolism, and immune regulation (23–25) (**Table 1**).

**Table 1:** ETS factors in cancer.

Subfamily	Gene name	Cancer	Refs
ETS	ETS1	Breast cancer; glioblastoma; colorectal cancer; clear cell renal cell carcinoma; ovarian cancer; gastric carcinoma; pancreatic carcinoma; thyroid cancer; esophageal cancer; lung cancer; hepatocellular carcinoma	(43–52)
	ETS2	Bladder cancer; hypopharyngeal cancer; prostate cancers; gastric cancer; esophageal squamous cell carcinoma; colon cancer	(53–57)
ERG	ERG	Glioblastoma; cervical cancer; prostate cancer; cholangio carcinoma; Ewing sarcoma	(58–64)
	FLI1	Bladder cancer; breast cancer; Ewing sarcoma; nasopharyngeal carcinoma	(65–68)
	FEV	Colorectal cancer; Ewing sarcoma	(69, 70)
ERF	ERF	Prostate cancer; bladder cancer	(71)
PEA3	ETV1	Hepatocellular carcinoma; Adeno carcinomas; prostate cancer; renal cell; Ewing sarcoma	(4, 63, 72–75)
	ETV4	Melanoma; Ewing sarcoma; hepatocellular carcinoma; endometrial cancer	(75–78)
	ETV5	Ewing Sarcoma; breast cancer; esophagus carcinoma; thyroid cancer; cervical cancer; endometrioid endometrial carcinoma	(75, 79–82)
TCF	ELK1	Hepatocellular carcinoma; endometrial cancer; thyroid cancer; cervical cancer	(83–86)
	ELK3	Glioma; hepatocellular carcinoma; breast cancer; cervical cancer	(87–89)
	ELK4	Cervical cancer; hepatocellular carcinoma	(90, 91)
ELG	GABPα	Prostate cancer; bladder cancer	(25, 92)
TEL	ETV6	Hematological malignancies; glioblastoma	(93, 94)
	ETV7	Breast cancer; bladder cancer	(95)
ELF	ELF1	Cervical cancer; osteosarcoma; prostate cancer; ovarian cancer	(63, 96–99)
	ELF2	Nasopharyngeal carcinoma; clear cell renal cell carcinoma; ovarian cancer; papillary thyroid carcinoma	(100–103)
	ELF4	Clear cell renal cell carcinoma; glioma; esophageal squamous cell carcinoma; hepatocellular carcinoma	(104–107)
SPI	SPI1	Lung squamous cell carcinoma; clear cell renal cell carcinoma; cervical cancer	(108–110)
	SPIB	Ovarian cancer; hepatocellular carcinoma	(42, 111)
ESE	EHF	Papillary thyroid carcinoma; cervical cancer; ovarian cancer	(112–114)
	ELF3	Bladder cancer; cervical cancer; ovarian cancer; gastrointestinal cancer;	(114)
	ELF5	Gastrointestinal cancer; endometrial carcinoma; renal cell carcinoma; ovarian cancer	(114–117)
PDEF	SPDEF	Neck squamous cell carcinoma; breast cancer; colorectal cancer; hepatocellular carcinoma; prostate cancer	(118–122)

Friend leukemia integration 1 transcription factor (FLI1); Fifth Ewing variant (FEV); ETS2 repressor factor (ERF); ETS variant 1(ETV1); ETS variant 4(ETV4); ETS variant 5(ETV5); ETS domain-containing protein Elk1(ELK1); ETS transcription factor ELK3 (ELK3); ETS domain-containing protein Elk-4 (ELK4); ETS variant transcription factor 7 (ETV7); E74-like ETS transcription factor 1 (ELF1); E74-like ETS transcription factor 2 (ELF2); E74-like ETS transcription factor 4 (ELF4); Spleen focus forming virus proviral integration oncogene (SPI1); Spi-B transcription factor (SPIB); Ets homologous factor (EHF); E74-like factor 3 (ELF3); E74-like factor 5 (ELF5); SAM pointed domain-containing Ets transcription factor (SPDEF).

### 2.2 Binding mechanism of ETS domain to DNA

The ETS domain is the core functional region of ETS transcription factors, showing a significant degree of conservation across different family members, particularly at the DNA-binding interface (26). This conservation enables ETS proteins to recognize and bind specific DNA sequences, thereby regulating the expression of downstream target genes. However, subtle variations in the sequence, domain length, and combination with other functional domains result in diverse DNA-binding specificities and transcriptional regulation capabilities among different ETS proteins (27).

The interaction between ETS proteins and DNA is a complex process involving multiple molecular interactions. The ETS domain typically recognizes and binds to the ETS binding site, usually a GGAA/T core sequence, located in the promoter or enhancer regions of target genes, through an α-helix-turn-α-helix motif (28–30). During this binding process, specific amino acid residues in the ETS domain form hydrogen bonds and hydrophobic interactions with DNA bases, stabilizing the protein-DNA complex (31, 32). Moreover, ETS proteins may interact with other transcription factors, further influencing transcriptional regulation (33, 34).

### 2.3 Regulatory network of ETS family proteins

The activity of ETS family proteins is not only regulated by external signals and other transcription factors but also by intrinsic molecular mechanisms. Post-translational modifications such as autophosphorylation and ubiquitination influence ETS protein stability, subcellular localization, and transcriptional activity, with these modifications catalyzed by specific enzymes and finely controlled by intracellular and extracellular signals (26, 35, 36).

ETS proteins also engage in combined regulation with other transcription factors. In complex gene expression networks, they often form transcription factor complexes that co-regulate target gene expression, enhancing regulatory specificity and efficiency. This coordinated regulation allows for fine-tuned transcriptional responses to different external signals and cellular states (37). Furthermore, ETS protein activity is modulated by growth factors and signaling pathways. For instance, epidermal growth factor (EGF) and fibroblast growth factor (FGF) activate the mitogen-activated protein kinase (MAPK) pathway, leading to phosphorylation and activation of ETS proteins (38, 39). Further, ETS-1 can inhibit transforming growth factor alpha (TGF-α) expression, thereby restoring tumor cell proliferation *in vivo* and promoting autonomous growth in culture (40). Other pathways, including Phosphoinositide 3-kinase (PI3K)/Protein Kinase B (Akt) and Janus kinase and signal transducer and activator of transcription (JAK-STAT), also contribute to ETS protein regulation by modulating phosphorylation, stability, and interactions with other molecules (41, 42).

## 3 The molecular characteristics and functions of ETS-1

### 3.1 Molecular structure of ETS-1

ETS-1, a key member of the ETS transcription factor family, has unique and complex structural characteristics. Its amino acid sequence is highly conserved, particularly in the ETS domain, which is essential for recognizing and binding DNA target sequences. In addition to the ETS domain, ETS-1 contains other functional regions, including a transcriptional activation domain, inhibition domain, nuclear localization signal (NLS), and potential phosphorylation sites. These domains collectively determine the transcriptional regulation activity and intracellular localization of ETS-1 (1, 123).

**Figure 1:**
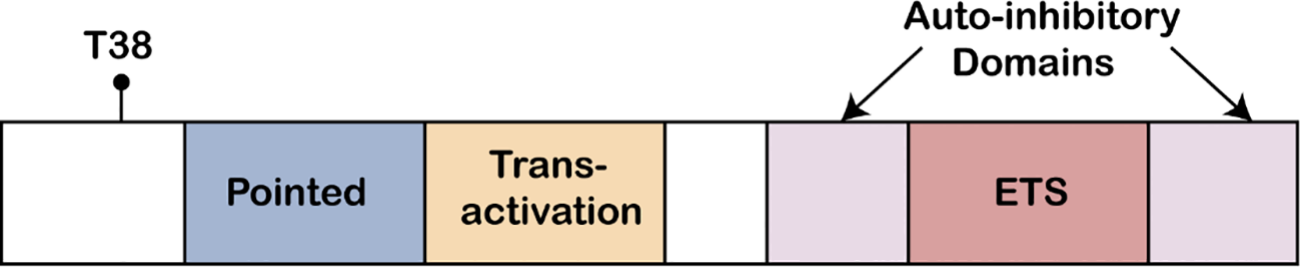
ETS protein structure. The ETS1 Pointed domain, the acidic transactivation domain, the autoinhibitory domains, and the Ets DNA binding domain, are indicated. Also shown is the conserved MAP kinase phosphorylation site (T38) found at the N-terminus of ETS1 protein.

The ETS domain is the core structure of ETS-1, having a unique α-helix-turn-α-helix DNA-binding motif (**Figure 1**). This domain relies on hydrogen bonds and hydrophobic interactions to perform its function (124). The high conservation of the ETS domain ensures the precise recognition and binding to specific ETS binding sites, such as the GGAA/T core sequence, thereby regulating the expression of downstream target genes (125).

### 3.2 Biological function of ETS-1

ETS-1 plays a crucial regulatory role in cell proliferation, differentiation, and migration (126). It influences gene expression by binding directly to the promoter or enhancer regions of target genes, thus affecting cell growth and differentiation (127). For example, ETS-1 can up-regulate cyclin expression, accelerating the transition from the G1 to S phase, thereby promoting cell proliferation (128). Moreover, ETS-1 is also involved in cytoskeleton remodeling and regulates the expression of intercellular adhesion molecules, impacting cell migration and invasion (129).

ETS-1 also modulates apoptosis and angiogenesis (130, 131). It regulates apoptosis by influencing the expression of genes such as Bcl-2 family members and caspases (132). Furthermore, ETS-1 is involved in regulating the expression of angiogenic factors, including vascular endothelial growth factor (VEGF) and fibroblast growth factor (FGF), which are critical for angiogenesis and tumor-related vascular development (133, 134). Therefore, abnormal ETS-1 expression is associated with various diseases characterized by dysregulated angiogenesis, such as cancer and diabetic retinopathy (44, 127, 130).

The expression of ETS-1 is both widespread and tissue-specific. While it is expressed to varying degrees in most normal tissues and organs, its levels are highly regulated temporally and spatially. During embryonic development, ETS-1 is essential for the formation of key organs, such as the heart, blood vessels, and nervous system (130, 135). In adult tissues, its expression is associated with physiological processes, including cell proliferation, differentiation, and immune responses (51, 132, 136). Under pathological conditions, such as in tumor tissues, ETS-1 expression is often abnormally elevated, correlating with its role in tumorigenesis, progression, and metastasis (47, 137).

## 4 ETS-1 and tumor immunity

### 4.1 Expression and regulation of ETS-1 in tumor

The expression of ETS-1 varies across different tumor types, with notable overexpression in several solid tumors, including breast cancer (11), prostate cancer (138), lung cancer (137), and colorectal cancer (45). Its expression levels also show significant changes in hematological malignancies, such as leukemia (139) and lymphoma (140). These variations may be attributed to the genetic background, the microenvironment of different tumors, and the functional specificity of ETS-1 in various cell types.

ETS-1 contributes to tumorigenesis and progression through multiple molecular mechanisms. It can directly regulate genes involved in cell proliferation, apoptosis, migration, and invasion, thereby promoting the malignant transformation and invasiveness of tumor cells (141, 142). The up-regulation of Ets-1 expression is intimately linked to tumour invasion and metastasis. It triggers the expression of a series of genes that have a crucial role in extracellular matrix remodeling and angiogenesis through transcription, and takes part in numerous invasion and metastasis processes such as extracellular matrix degradation and cell migration (142). Furthermore, ETS-1 may maintain the sustained growth and metastasis of tumor tissues by modulating the self-renewal and differentiation of cancer stem cells (143).

### 4.2 The effect of ETS-1 on tumor immune microenvironment

ETS-1 plays a crucial role in shaping the tumor immune microenvironment by regulating immune cell infiltration, activation, and the expression of immunosuppressive factors. It influences the migration and infiltration of immune cells into tumor tissues by modulating chemokines (e.g., CXCL12) and adhesion molecules (e.g., ICAM-1) (144), and affects immune cell activation and anti-tumor responses by regulating the expression and function of immune cell surface receptors (145). Furthermore, ETS-1 promotes tumor immune escape by directly regulating the expression of multiple immune checkpoint molecules. While the exact role of ETS-1 in the upregulation of PD-L1, CTLA-4, LAG-3, and TIM-3 requires further confirmation, studies suggest that ETS-1 may contribute to the regulation of these molecules, which are known to suppress T cell activation and function, thus enabling tumor cells to evade immune recognition and clearance (123, 146, 147). Consequently, the pivotal role of ETS-1 in tumor immune evasion highlights its potential as a promising target for immunotherapy.

As a multifunctional transcription factor, ETS-1 regulates genes associated with immunosuppression, thereby facilitating tumor immune escape. For example, ETS-1 exerts a pivotal influence in renal epithelial cells, particularly in the context of TGF-β1 stimulation. It primarily suppresses the downstream effects of the TGF-β signaling pathway, which is indicative of its significant regulatory role in extracellular matrix remodeling and fibrosis. This regulation is likely mediated through its impact on key molecules within the TGF-β signaling cascade. Additionally, ETS-1 plays a crucial role in shaping the tumor immune microenvironment by regulating the expression of immunosuppressive factors, such as TGF-β. TGF-β is known to inhibit T cell activation and proliferation, while enhancing the function of regulatory T cells (Tregs) and myeloid-derived suppressor cells (MDSCs), thus contributing to immune evasion. While the exact mechanism of ETS-1 activation of TGF-β expression requires further validation, studies suggest that ETS-1 may regulate TGF-β and its associated immune responses, underscoring its complex role in both fibrosis and immune modulation (148–150). ETS-1 also regulates interleukin-10 (IL-10), a cytokine that suppresses immune cell function by inhibiting antigen presentation by macrophages and dendritic cells and reducing the production of Th1 cytokines (151, 152). These factors contribute to immune escape through complex signaling pathways and cell-cell interactions, allowing tumor cells to evade the recognition and clearance of the immune system.

ETS-1 plays a pivotal role in the tumour immune microenvironment by modulating antigen presentation and cytokine networks through multiple mechanisms. Research has demonstrated that ETS-1 regulates the expression of MHC Class I molecules in tumour cells, thereby influencing the recognition of tumour cells by cytotoxic T cells (153). Additionally, ETS-1 modulates the expression of cell adhesion molecules, such as ICAM-1, which affects the infiltration of immune cells into the tumour microenvironment (154). These actions not only enhance the immune evasion capabilities of tumour cells but also weaken antitumor immune responses by influencing the adhesion and migration of immune cells. Furthermore, ETS-1 is involved in the regulation of various cytokines, including TGF-β and IL-10, which inhibit antigen presentation and reduce the production of Th1 cytokines, thereby further suppressing immune responses (151). Collectively, these studies highlight the multifaceted role of ETS-1 in promoting tumour immune evasion and progression by regulating antigen presentation and cytokine networks.

In addition to its direct effects on immune cells, ETS-1 influences the tumor microenvironment by regulating the extracellular matrix (ECM) composition and structure. The ECM plays a critical role in tumor growth, invasion, and metastasis, and ETS-1 contributes to ECM remodeling by up-regulating matrix metalloproteinases (MMPs) (155–157). MMPs are a class of enzymes that degrade various extracellular matrix components, and they play a key role in the migration and invasion of tumor cells (158). ETS-1 up-regulates the expression of MMPs, which not only helps tumor cells break through the matrix barrier and infiltrate and metastasize to surrounding tissues but also affects the infiltration and distribution of immune cells by changing the physical structure of the tumor microenvironment (159). For example, ECM degradation by MMPs may enhance vascular permeability, allowing more immune cells and inflammatory factors to enter the tumor microenvironment (160, 161). Structural changes in the ECM can also impact immune cell movement and distribution patterns, influencing anti-tumor immune responses (162–164).

### 4.3 ETS-1 related signaling pathway

**Figure 2:**
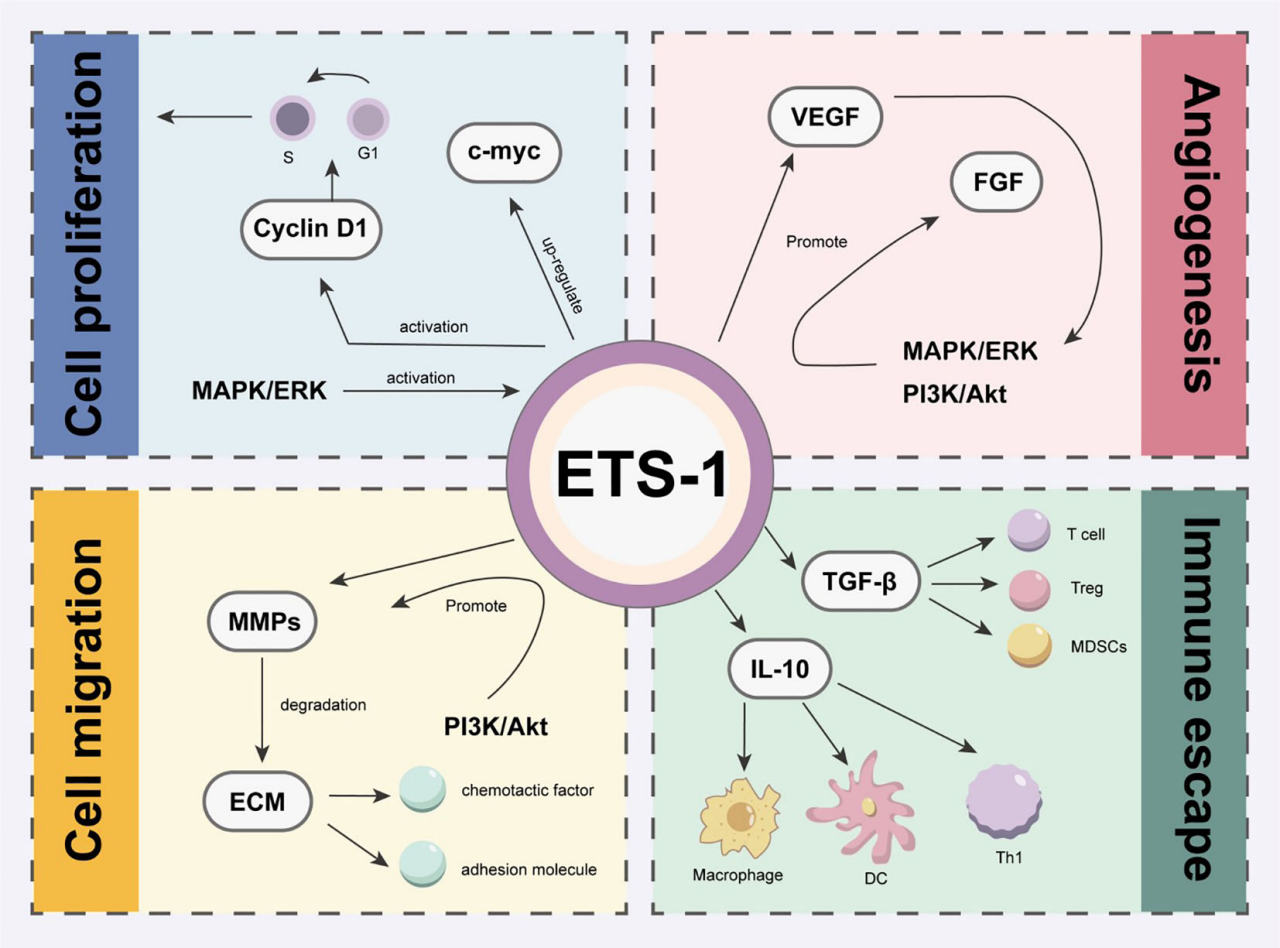
The regulatory effect of ETS-1 related signaling pathway on tumor biological processes. This figure shows the role of ETS-1 in cell proliferation, cell migration, immune escape and angiogenesis through the three main signaling pathways of MAPK/ERK, PI3K/Akt and TGF-β.

ETS-1 plays a significant regulatory role in various biological processes of tumors, with its function being influenced by multiple signalling pathways, particularly the MAPK/ERK, PI3K/Akt, and TGF-β pathways. Firstly, ETS-1 is a key target of the MAPK/ERK (Mitogen-Activated Protein Kinase/Extracellular Signal-Regulated Kinase) pathway, which is crucial for cell proliferation, differentiation, survival, and response to external stimuli. Secondly, the expression and activity of ETS-1 are also regulated by the PI3K/Akt (Phosphoinositide 3-Kinase/Protein Kinase B) pathway. This pathway, essential for cell survival and metabolism, stabilizes ETS-1 protein and promotes its nuclear accumulation, thereby enhancing its transcriptional function. Additionally, the TGF-β (Transforming Growth Factor Beta) pathway, under the regulation of ETS-1, affects the remodeling of the extracellular matrix and the infiltration of immune cells. TGF-β signalling promotes ETS-1 expression, influencing the immune response within the tumour microenvironment and the potential for tumour metastasis, thereby supporting the aggressiveness of tumors (**Figure 2**).

## 5 The role of ETS-1 in specific tumor types

### 5.1 ETS-1 in breast cancer

In breast cancer, high expression of ETS-1 is closely associated with poor prognosis, increased malignancy, and invasiveness. Studies indicate that elevated ETS-1 levels correlate with higher tumor grade, greater risk of lymph node metastasis, and shorter patient survival (11, 127, 165). Thus, ETS-1 expression may serve as a valuable prognostic biomarker for breast cancer.

ETS-1 plays a key role in regulating breast cancer cell proliferation and metastasis. It acts as a transcription factor by binding to the promoter regions of cell cycle-related genes, such as *cyclin D1* and *c-myc*. *Cyclin* D1, a key regulator in the cyclin family, is crucial for the transition from the G1 to S phase during cell division. ETS-1 directly activates *cyclin D1* transcription by binding to its promoter, resulting in increased *Cyclin* D1 protein levels, which accelerate the progression through the G1/S checkpoint and increase breast cancer cell proliferation. Similarly, ETS-1 up-regulates the proto-oncogene *c-myc*, a transcription factor involved in cell proliferation, differentiation, and apoptosis. By increasing *c-myc* gene transcription, ETS-1 increases the accumulation of *c-Myc* protein, a nuclear-phosphorylated protein with transcription factor activity, thereby activating downstream targets such as ribosomal biosynthesis and cell cycle regulatory genes. This creates a positive feedback loop that further promotes cancer cell proliferation (166–169).

### 5.2 ETS-1 in gastric cancer

ETS-1 plays a significant role in the development and progression of gastric cancer. Research indicates that ETS-1 expression is significantly elevated in gastric cancer tissues compared to normal gastric mucosa and is strongly associated with the pathological stage, lymph node metastasis, and patient prognosis (170). ETS-1 contributes to gastric cancer progression by promoting cell proliferation, inhibiting apoptosis, and facilitating migration and invasion (171). An important mechanism in gastric cancer progression is immune escape, which enables tumor cells to evade immune surveillance and destruction. Inhibiting ETS-1 expression or function may help disrupt the immune escape process, potentially restoring the anti-tumor activity of the immune system (56, 142).

### 5.3 ETS-1 in other tumor types

ETS-1 plays a significant regulatory role in colorectal cancer, where its high expression is strongly associated with increased malignancy, lymph node metastasis, and distant metastasis (166, 172). It promotes the occurrence and development of colorectal cancer by regulating cell proliferation, migration, and invasion (173). Furthermore, ETS-1 can induce epithelial-mesenchymal transition (EMT), increasing the invasive and migratory capabilities of colorectal cancer cells, thereby facilitating metastasis (174). ETS-1’s interaction with immune cells and matrix components in the tumor microenvironment affects local inflammation and immune evasion, further contributing to colorectal cancer progression. Thus, ETS-1 serves as both a key regulator in colorectal cancer biology and a potential therapeutic target. Targeting ETS-1 could provide new treatment options and improve clinical outcomes for patients.

In prostate cancer, ETS-1 expression is often higher compared to normal prostate tissue, correlating with disease progression and prognosis (175). ETS-1 may interact with multiple signaling pathways, including the androgen receptor (AR) pathway, which is important in androgen-dependent prostate cancer (176). Moreover, ETS-1 may interact with other transcription factors, such as Nuclear Factor Kappa B (NF-κB), modulating inflammation and the tumor microenvironment (177).

In addition, ETS1 can reduce the mRNA levels of Twist1, a gene involved in tumor cell motility and dissemination, thereby decreasing invasion and increasing cell growth to mitigate lung tumor metastasis. Downregulation of the ETS1 gene can inhibit cell proliferation and migration in non-small cell lung cancer (NSCLC) cells, slowing the progression of lung cancer (51, 178). In chronic myeloid leukemia (CML), ETS1 is associated with the BCR-ABL signaling pathway, regulates granulocyte differentiation, and is downregulated during tyrosine kinase inhibitor (TKI) treatment (179). In diffuse large B-cell lymphoma (DLBCL), ETS1 modulates B-cell signaling, differentiation, and immune-related genes, with FCMR identified as a novel target that promotes lymphomagenesis. In adult T-cell leukemia/lymphoma (ATLL), ETS1 is highly expressed in patients of North American descent and promotes tumor growth by regulating CCR4, making it a potential therapeutic target (180, 181).

**Figure 3:**
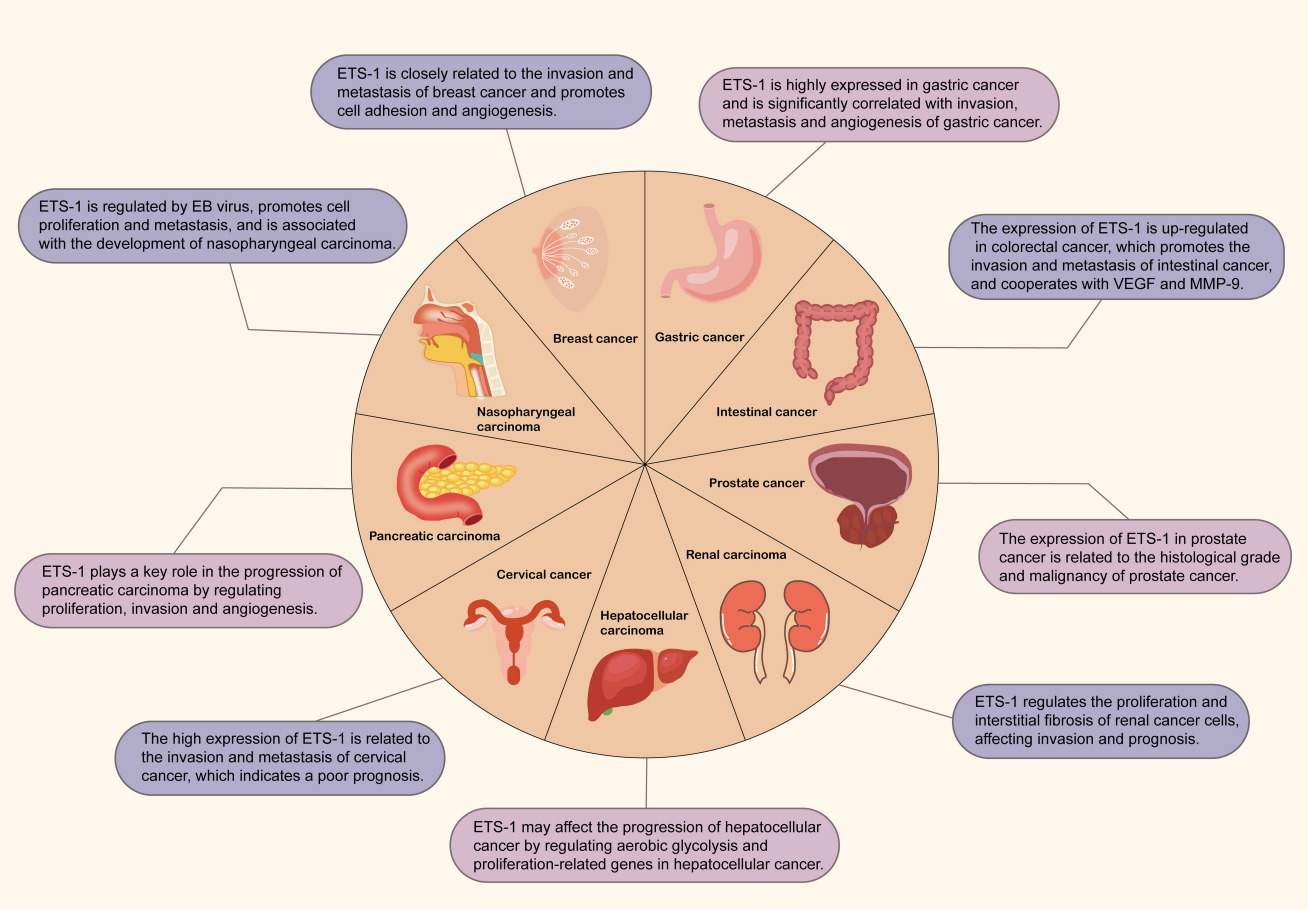
The expression of ETS-1 in different tumors. The map contains the role of ETS1 in breast cancer, gastric cancer, colorectal cancer, prostate cancer, kidney cancer, liver cancer, cervical cancer, pancreatic cancer and nasopharyngeal carcinoma.

Overall, ETS-1 plays a key regulatory role across various tumor types, with its expression and functional activity closely associated with tumor occurrence, development, prognosis, and response to immunotherapy. Therefore, understanding the mechanisms of ETS-1 in cancer is of great significance for developing novel anti-tumor strategies (**Figure 3**).

## 6 The potential of ETS-1 as a therapeutic target

With an in-depth understanding of the mechanism of action of ETS-1 in tumors, the development of inhibitors for ETS-1 has become a research focus. Current approaches to inhibiting ETS-1 include small molecule compounds, antisense oligonucleotides (ASOs), and CRISPR/Cas9-based gene editing tools. Small molecule compounds inhibit ETS-1’s transcriptional activity by binding to its DNA-binding or transcriptional activation domains (10). ASOs (antisense oligonucleotides) may interfere with the expression of ETS-1 by binding to its mRNA, thereby blocking its translation. Although the precise mechanism is not fully established in the current literature, the role of antisense oligonucleotides in regulating the expression of transcription factors has been widely studied, suggesting that they may influence the regulation of ETS-1 (182, 183). CRISPR/Cas9-based gene editing can knock out the *ETS-1* gene, effectively eliminating its expression (164, 184). While their mechanisms differ, these inhibitors share a common goal of reducing ETS-1 expression and activity in tumor cells.

Chimeric antigen receptor T cell (CAR-T) therapy, an emerging immunotherapeutic approach, utilizes genetically engineered T cells to target and destroy tumour cells (185–187). ETS-1, an intracellular transcription factor, is a key player in tumorigenesis, making it a potential target for CAR-T therapies. However, targeting intracellular proteins like ETS-1 is challenging due to the traditional focus of CAR-T therapies on cell surface antigens. Recent advances, such as CRISPR-Cas9 gene editing and novel vector systems, have enabled the design of CAR-T cells capable of targeting specific intracellular molecules (188, 189). Additionally, research is exploring adapter molecules and logic gate designs to enhance CAR-T specificity and activity (190). While these innovations offer potential for targeting ETS-1, further optimization in design, production, and clinical application is needed. Future directions may include developing CAR structures that can penetrate the cell membrane or modulating ETS-1 expression through pharmacological and genetic approaches to enhance therapy efficacy.

Although ETS-1 inhibitors remain in preclinical development, preliminary data suggest their potential therapeutic value. In animal models, ETS-1 inhibitors have demonstrated significant suppression of tumor growth and metastasis, along with enhancement of anti-tumor immune responses (10, 52). However, clinical translation faces challenges, including issues of specificity, efficacy, safety, and optimal administration routes. Future clinical trials must focus on evaluating the safety, tolerability, and efficacy of ETS-1 inhibitors in humans to assess their potential as viable cancer therapeutics.

## 7 Conclusions and prospects

This review highlights the pivotal role of ETS-1 in tumor immunity, detailing its aberrant expression across various tumor types and its significant influence on tumorigenesis, progression, immune evasion, and sensitivity to immunotherapy. ETS-1 promotes tumor advancement not only by regulating tumor cell proliferation, migration, and invasion but also by contributing to immune escape mechanisms through modulation of immune cell infiltration, activation, and immune checkpoint molecule expression within the tumor microenvironment. These findings offer a new perspective on the molecular basis of tumor immunity and present ETS-1 as a promising target for developing novel anti-cancer therapies.

The clinical potential of ETS-1 in tumor immunotherapy is vast. As research into the functional mechanisms of ETS-1 progresses and therapeutic technologies advance, ETS-1 could emerge as a key target for cancer immunotherapy. In clinical settings, assessing ETS-1 expression levels may provide a means to evaluate patient prognosis and guide personalized treatment decisions. Moreover, developing targeted therapies and immunotherapeutic strategies directed at ETS-1 could offer new therapeutic options and improve outcomes for cancer patients.

To fully realize the potential of ETS-1 as a therapeutic target and facilitate its clinical translation, future research should prioritize the following areas: First, a deeper investigation into the molecular mechanisms of ETS-1 in tumorigenesis, including its interactions with key molecules and signaling pathways, is essential. Second, efforts should be directed toward developing more specific and efficacious ETS-1 inhibitors while minimizing potential toxicities. Third, exploring the combined use of ETS-1-targeted therapies with other immunotherapeutic strategies, such as immune checkpoint inhibitors or CAR-T cell therapy, could achieve synergistic therapeutic effects. Fourth, attention must be given to understanding and overcoming drug resistance mechanisms that may arise during ETS-1-targeted treatment. Finally, conducting large-scale clinical trials is crucial to validate the efficacy and safety of ETS-1-targeted therapies, thus providing robust evidence for clinical application.
